# Radiation Exposure to the Brains of Interventional Radiology Staff: A Phantom Study

**DOI:** 10.3390/bioengineering11111083

**Published:** 2024-10-29

**Authors:** Saya Ohno, Ryota Shindo, Satoe Konta, Keisuke Yamamoto, Yohei Inaba, Koichi Chida

**Affiliations:** 1 Course of Radiological Technology, Health Sciences, Tohoku University Graduate School of Medicine, 2-1 Seiryo, Aoba-ku, Sendai 980-8575, Japan; saya.ono1223@gmail.com (S.O.); ryota.shindo.r5@dc.tohoku.ac.jp (R.S.); satoe.konta.q8@alumni.tohoku.ac.jp (S.K.); keisuke.yamamoto.r3@dc.tohoku.ac.jp (K.Y.); inabay@tohoku.ac.jp (Y.I.); 2 Department of Radiological Disasters and Medical Science, International Research Institute of Disaster Science (IRIDeS), Tohoku University, 468-1 Aramaki Aza-Aoba, Aoba-ku, Sendai 980-0845, Japan

**Keywords:** X-ray examination, medical radiation dose, interventional radiology (IR), radiation safety, occupational exposure dose, radiation protection and shielding, radiation injuries (cataracts and brain tumor), radioprotective cap, disaster medicine, eye radiation exposure

## Abstract

Numerous papers report the occurrence of head and neck tumors in interventional radiology (IR) physicians. Recently, appropriate dosimetry and protection have become much more important. To accomplish these, first, we should accurately understand how the brain is exposed. We assessed the dose distribution of the head and clarified the relationship between head exposure and brain dose. We used eight radiophotoluminescence dosimeters (RPLDs), two at the surface of the eyes and six inside the phantom head. We conducted measurements with three kinds of irradiation fields: one irradiated the whole head, the second irradiated the brain region, and the third irradiated the soft tissue of the face. The cranial bone reduced the brain dose to less than half the skin dose: about 48% at the front and less than 9% at the back of the brain. Due to the brain exposure, the soft tissues were slightly exposed to the scatter radiation from the cranial bone. We revealed the dose distribution of the head and the influence of the scatter radiation from the cranial bone and the soft tissues of the face. There are two kinds of scatter radiation: from the cranial bone to the soft tissue of the face, and from the soft tissue to the brain. Although the influence of these sources of scatter radiation is not significant, the relationship between brain exposure and the occurrence of head and neck tumors is still unclear. Therefore, some IR physicians should keep this in mind if they receive high levels of exposure in their daily practice.

## 1. Introduction

The practice of radiology has been part of medicine for a long time [1,2,3,4]. While many patients have benefited from the many advances in the field, the medical exposure of patients and the occupational exposure of medical staff to radiation has increased [5,6,7,8,9,10]. In particular, the number of interventional radiology (IR) procedures has increased. During IR procedures, not only patients but also IR physicians sometimes receive high levels of radiation exposure [11,12,13,14,15,16]. Thus, some of them have suffered from various radiation injuries, such as skin erythema and cataracts [17,18,19,20,21,22].

Numerous epidemiological studies have assessed the association between the occupational radiation exposure of medical staff and brain tumor risk. However, there is little convincing evidence of increased head tumor risk caused by occupational radiation exposure [23,24,25,26,27,28]. Rajaraman found an approximately two-fold increased risk of brain cancer for radiological technologists who perform fluoroscopy-guided interventional procedures; however, Rajaraman mentioned the possibility of other confounding factors [29]. Some researchers have reported head and neck tumors occurring in IR physicians [30,31]. Roguin et al. [30] reported, in their paper about the head and neck tumors of IR physicians, cardiologists, and radiologists, that 55% of tumors were glioblastomas, and 85% of those occurred in the left brain.

As described above, it is still unclear and controversial whether there is a relationship between the occurrence of head tumors and radiation exposure at low levels. Considering that the International Commission on Radiological Protection (ICRP) regards the threshold dose as 0.5 Gy, above which there is likely an increased risk of cardiovascular and cerebrovascular disease [32], it is quite important to protect physicians’ heads adequately. Furthermore, it is thought that backscatter radiation generated from the head contributes to the eye lens dose [33]. Hence, head protection would help with eye lens protection.

IR staff must protect not only their trunk but also their head and neck, so diverse radioprotective equipment, such as a ceiling-suspended lead screen, a lead apron, and protective eyewear, has been employed. Numerous studies, both phantom studies and clinical research, have evaluated the efficacy of this radioprotective equipment [34,35,36,37,38,39,40,41,42,43]. Additionally, a novel radiation shield for the face and neck was developed recently [44]. This novel radiation shield, TRIPLE GUARD (Maeda & Co., Ltd., Tokyo, Japan), has both a neck guard and a face shield. Since its face shield is specifically designed to protect the left side of the face, IR physicians can reduce the dose to their face efficiently. Sometimes, IR physicians also use the lead-free cap to protect their heads. The effectiveness of the radioprotective cap is controversial as several studies argue that the radioprotective cap is effective for head protection [45,46], but other studies found that the radioprotective cap is less effective [47,48,49].

To best assess and develop appropriate IR physician head protection, we should accurately understand how the brain is exposed. Therefore, in this study, we evaluated the radioprotective effect of the brain and scatter radiation generated from the head using an anthropomorphic phantom.

## 2. Materials and Methods

This study used radiophotoluminescence dosimeters (RPLDs) made of silver-activated phosphate glass (GD-302M, Chiyoda Technol Corporation, Tokyo, Japan). We used the measurement/readout system, Dose Ace FDG-1000 (Chiyoda Technol Corporation, Japan). Before we started the measurements, RPLDs were annealed (400 °C, 20 min) to reset the accumulated dose. After the measurements were completed, we preheated the RPLDs to stabilize the luminescence.

The RPLDs have energy dependencies, resulting in their increased sensitivities being in the lower-energy ranges. To compensate for this, we performed the following steps: first, we determined the effective energy from the half-value layer of aluminum [50], and then we used a formula [51] to calculate the calibration factor from the effective energy, and finally, we divided each measurement by this calibration factor.

**Figure 1:**
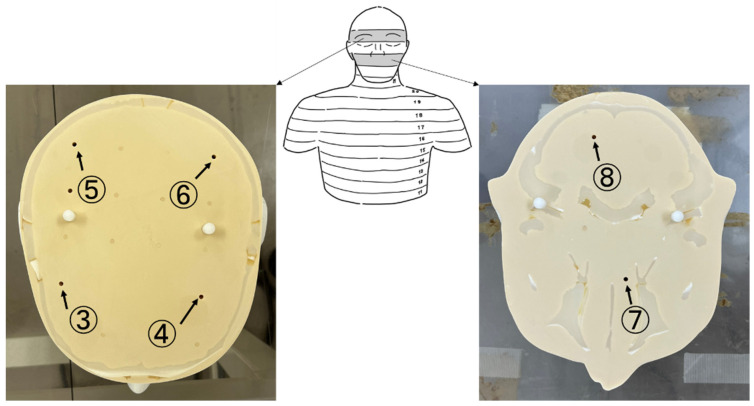
Photographs showing where the radiophotoluminescence dosimeters (RPLDs) were placed in each slice. Slice No. 25 had four RPLDs in four holes (③, ④, ⑤, ⑥). Slice No. 23 had two RPLDs in two holes (⑦, ⑧).

We used an anthropomorphic phantom (THRA1, Kyoto Kagaku, Kyoto, Japan) where each slice can be separated. As shown in Figure 1, we used eight RPLDs in one measurement: six inside the phantom and one for each eye.

**Figure 2:**
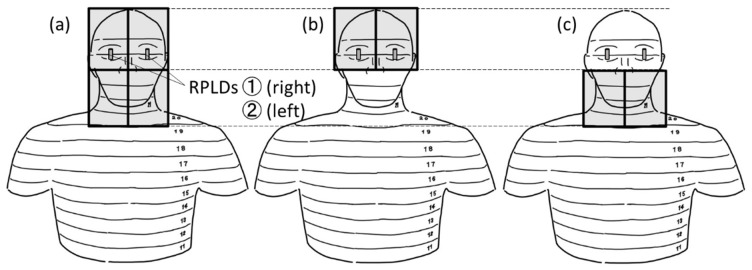
Illustrations of the three kinds of radiation fields. (**a**) Field 1 covers the whole head and neck of the phantom. (**b**) Field 2 covers the brain of the phantom. The lower edge of this field is the upper edge of slice No. 23. (**c**) Field 3 covers the cerebellum and the soft tissues of the phantom’s face. The upper edge of this field is the upper edge of slice No. 23.

**Figure 3:**
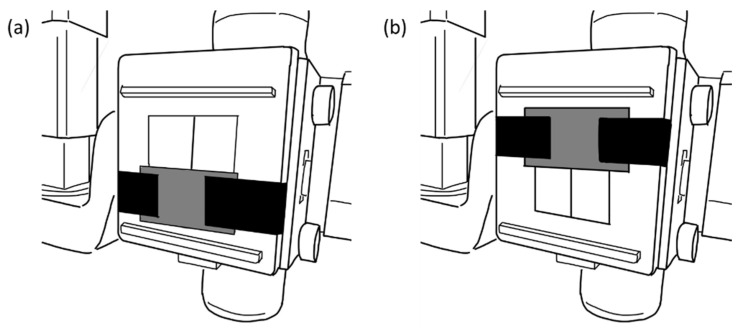
Illustrations that depict how Fields 2 and 3 were set using a lead plate. (**a**) For the measurements in Field 2, a lead plate was placed in the lower half of the exposure window. (**b**) For the measurements in Field 3, a lead plate was placed in the upper half of the exposure window.

The X-ray system used was DHF-155H II (HITACHI, Tokyo, Japan). The measurements were performed with the following settings: a tube voltage of 100 kV, a current intensity of 1 mA, and a fluoroscopy time of 60 s. We set irradiation fields (Figure 2): 26 × 19 cm (hereinafter Field 1) such that it covered the whole head and neck of the phantom, 13 × 19 cm (hereinafter Field 2) such that it covered the brain of the phantom, and 13 × 19 cm (hereinafter Field 3) such that it covered the cerebellum and the soft tissues of the phantom’s face. To guarantee repeatability during the measurements, we used a lead plate to cover half of the exposure window for the measurements in Fields 2 and 3 (Figure 3).

**Figure 4:**
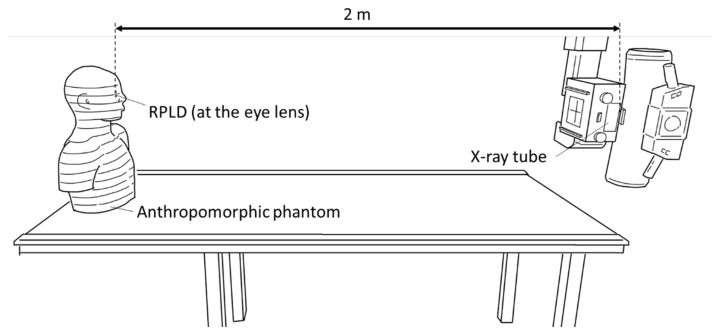
Geometry of the measurements. The distance between the source and the surface of the phantom’s eye was set to 2 m.

The geometry of the measurements is shown in Figure 4. The distance between the X-ray source and the surface of the phantom’s eye was set at 2 m.

We measured each irradiation field six times and calculated the mean ± standard deviation (SD).

## 3. Results

**Figure 5:**
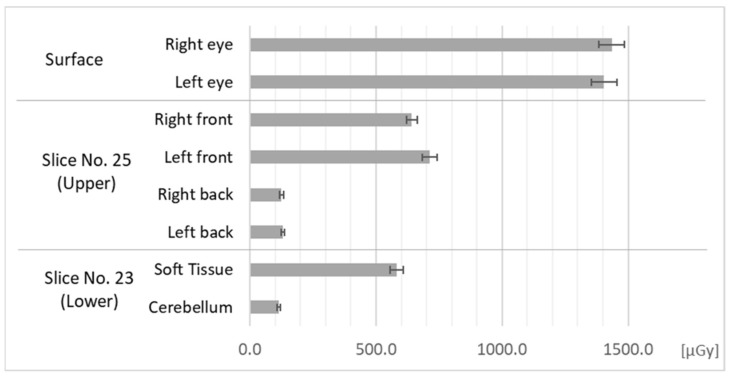
Doses at each measurement point in the phantom in Field 1.

Absorbed doses measured in Field 1 are shown in Figure 5. In the measurements of Field 1, we irradiated the whole head and neck of the phantom.

Doses at the front region of the brain were about 48% of those at the skin surface. Doses in the back region of the brain were about 9%. Both the soft tissue of the face and cerebellum received slightly lower doses than each corresponding region of the brain.

**Figure 6:**
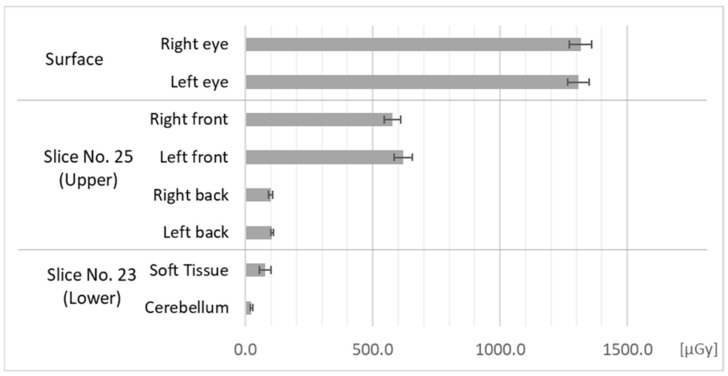
Doses at each measurement point in the phantom in Field 2.

**Figure 7:**
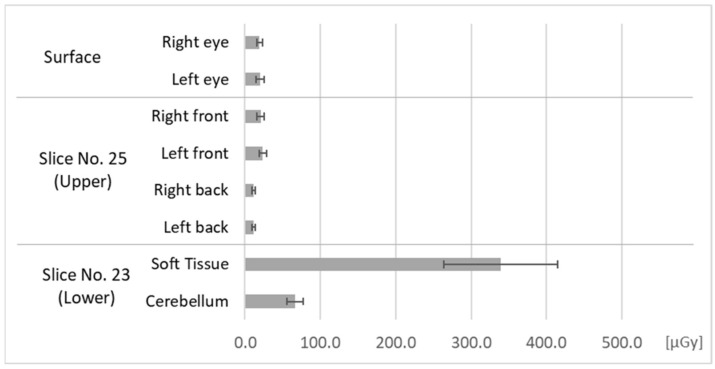
Doses at each measurement point in the phantom in Field 3.

Figure 6 and Figure 7 show absorbed doses measured in Fields 2 and 3. In the Field 2 measurements, we irradiated the upper bounds of the phantom’s face, mainly its brain. In the Field 3 measurements, we irradiated the lower bounds of the phantom’s face, mainly its soft tissues, which do not cover the cranial bone.

As seen in Figure 6, the soft tissues of the phantom’s face were slightly exposed by the exposure of the brain. In contrast, as seen in Figure 7, the brain was very slightly exposed by exposure of the soft tissues of the face, however, the doses were lower than the soft tissue dose in Figure 6.

## 4. Discussion

Since the number of IR procedures has increased, it is crucial to reduce medical and occupational radiation exposure [52,53,54,55,56,57,58,59]. Numerous papers have described the radiation injuries of both patients and IR staff [60,61,62,63,64,65,66], and some researchers have reported the possibility of IR physicians being at high risk of head and neck tumors [30,31,67,68]. In addition, the ICRP regards 0.5 Gy as an acute dose threshold for cardiovascular disease (including cerebrovascular disease). IR physicians need to understand their brain exposure and protect themselves appropriately.

This study demonstrated how much the cranial bone protects the brain. The cranial bone reduced the brain dose to less than half the skin dose: about 48% at the front and less than 9% at the back of the brain. Marsh [69] contends that the cranial bone absorbs the scatter radiation at about 40%, and our study supports this.

Referring to the previous studies, we compared the protective efficiency between the cranial bone and the protective equipment. The protective eyewear, made of 0.07 mm Pb-equivalent, has a protective efficiency of 61.4% [39], while the radioprotective surgical cap, made of 0.06 mm Pb-equivalent, has an efficiency of 71% [48]. Considering that the protective efficiency of the cranial bone to the front region of the brain was about 48% in our measurement, the cranial bone has enough radioprotective capability, though to a lesser extent than the radioprotective equipment. Although Endo [39] reported a protective efficiency of 61.4% for the eye lens dose, it is important to note that the eye lens does not contain bone. The protection in that study is provided by a lead–acrylic transparent material in front of the eye lens [39]. On the other hand, Kirkwood [48] examined the protective efficiency of the RADPAD No Brainer surgical cap, which is made of non-transparent material. It demonstrates an attenuation of 71% ± 2.0% at the temporal position, likely due to lead or lead-equivalent material in the cap [48]. However, the study also shows that the eye lens dose remains unchanged (−1.5% ± 1.4%) because the protective cap was not applied to the same locations as in reference [39].

Additionally, studies on X-rays have shown that the shielding factor provided by standard protective eyewear is generally very small [70]. The presence of lead-equivalent materials could potentially alter this, but the protective efficiency between cranial bone and X-rays requires further investigation.

Since Lemesrse et al. [71] reported that the skin of the physicians’ heads is exposed to approximately 2 mSv/year, considering our results, the physicians’ brains might be exposed to less than 1 mSv/year. Considering it is still controversial whether there is a relationship between the occurrence of head tumors and radiation exposure, we recommend IR physicians wear radioprotective equipment for the head to guarantee their safety.

When we irradiated only the upper bounds of the phantom, mainly the brain, the soft tissues were slightly exposed because of the scatter radiation generated from the cranial bone. In this study, because we included the eye in the irradiation field, it is unclear whether there is a reliable relationship between the eye dose and the brain exposure. However, it is possible that the scatter radiation from the cranial bone would increase the eye dose. On the other hand, when we irradiated only the lower bounds of the phantom, mainly the soft tissues of the face not covered by the cranial bone, the brain was very slightly exposed. Considering that doses of the front part of the brain were slightly higher than the eye dose, the scatter radiation generated from the soft tissues of the face could slightly increase the brain dose, however, this contribution would be almost negligible.

The influence of both scatter radiations, from the cranial bone to the eye and from the soft tissues of the face to the brain, is not significant; however, IR physicians should be concerned about the cumulative effect in those who perform numerous procedures and receive high levels of occupational exposure.

Quality control (QC) and quality assurance (QA) programs are crucial for dose measurements. For instance, Canadian regulations, which are aligned with international standards, require an annual independent dose measurement test [72,73,74]. In this test, an external lab exposes dosimeters to an unknown dose and sends them to the dosimetry lab for estimation. The estimated results are then compared to the actual delivered dose to ensure the precision and reliability of the dose measurements [72,73,74]. Therefore, further consideration may be needed regarding an independent method (QC and QA programs) for estimating the uncertainty of the dose measurement process in our study.

### Limitation

In this study, we only used X-rays from the front of the phantom. However, in practice, IR physicians typically work with the left side of their heads close to the primary X-ray source and the patient, and the source of scatter radiation is below the IR physician’s front. Thus, IR physicians are typically exposed from the lower left. Because of this, the actual dose distribution of the head would differ from our results.

## 5. Conclusions

We revealed the dose distribution of the head and the influence of the scatter radiation from the cranial bone and the soft tissues of the face. The cranial bone reduced the brain dose to less than half. The scatter radiation generated from the cranial bone might slightly increase the exposure of the eye as well as the soft tissues of the face. On the other hand, the amount of scatter radiation generated from the soft tissues of the face is lower than the scatter radiation from the cranial bone. Although the influence of these sources of scatter radiation is not significant, the relationship between brain exposure and the occurrence of head and neck tumors is still unclear, so IR physicians who receive high levels of exposure in their daily practice should keep these sources of scatter radiation in mind.

## Data Availability

Data are contained within the article.

## Abbreviations

ICRP	International Commission on Radiological Protection
IR	interventional radiology
QA	quality assurance
QC	quality control
RPLD	radiophotoluminescence dosimeter
SD	standard deviation
